# A systematic review to determine the effect of strategies to sustain chronic disease prevention interventions in clinical and community settings: study protocol

**DOI:** 10.1186/s13643-024-02541-0

**Published:** 2024-05-09

**Authors:** Edward Riley-Gibson, Alix Hall, Adam Shoesmith, Luke Wolfenden, Rachel C. Shelton, Emma Doherty, Emma Pollock, Debbie Booth, Ramzi G. Salloum, Celia Laur, Byron J. Powell, Melanie Kingsland, Cassandra Lane, Maji Hailemariam, Rachel Sutherland, Nicole Nathan

**Affiliations:** 1 School of Medicine and Public Health, The University of Newcastle, Newcastle, NSW Australia; 2 Priority Research Centre for Health Behaviour, The University of Newcastle, Newcastle, NSW Australia; 3 Hunter Medical Research Institute, New Lambton Heights, NSW Australia; 4 Hunter New England Local Health District, Hunter New England Population Health, Newcastle, NSW 2287 Australia; 5 Department of Sociomedical Sciences, Mailman School of Public Health, Columbia University, New York, NY USA; 6 Division of Public Health, College of Human Medicine, Michigan State University, Flint, MI USA; 7 Department of Health Outcomes & Biomedical Informatics, University of Florida College of Medicine, Gainesville, FL USA; 8 Women’s College Hospital Institute for Health System Solutions and Virtual Care, Toronto, 76 Grenville StreetOntario M5S 1B2 Canada; 9 Institute of Health Policy, Management and Evaluation, University of Toronto, Health Sciences Building, 155 College Street, Suite 425, Toronto, ON M5T 3M6 Canada; 10 Center for Mental Health Services Research, Brown School, Washington University in St. Louis, St. Louis, MO USA; 11 Center for Dissemination and Implementation, Institute for Public Health, Washington University in St. Louis, St. Louis, MO USA; 12 Division of Infectious Diseases, John T. Milliken Department of Medicine, School of Medicine, Washington University in St. Louis, St. Louis, MO USA

**Keywords:** Sustainment, Sustainability, Strategies, Chronic isease revention

## Abstract

**Background:**

The primary purpose of this review is to synthesise the effect of strategies aiming to sustain the implementation of evidenced-based interventions (EBIs) targeting key health behaviours associated with chronic disease (i.e. physical inactivity, poor diet, harmful alcohol use, and tobacco smoking) in clinical and community settings. The field of implementation science is bereft of an evidence base of effective sustainment strategies, and as such, this review will provide important evidence to advance the field of sustainability research.

**Methods:**

This systematic review protocol is reported in accordance with the Preferred Reporting Items for Systematic review and Meta-Analysis (PRISMA) checklist. Methods will follow Cochrane gold-standard review methodology. The search will be undertaken across multiple databases, adapting filters previously developed by the research team, data screening and extraction will be performed in duplicate, strategies will be coded using an adapted sustainability-explicit taxonomy, and evidence will be synthesised using appropriate methods (i.e. meta-analytic following Cochrane or non-meta-analytic following SWiM guidelines). We will include any randomised controlled study that targets any staff or volunteers delivering interventions in clinical or community settings. Studies which report on any objective or subjective measure of the sustainment of a health prevention policy, practice, or programme within any of the eligible settings will be included. Article screening, data extraction, risk of bias, and quality assessment will be performed independently by two review authors. Risk of bias will be assessed using Version 2 of the Cochrane risk-of-bias tool for randomised trials (RoB 2). A random-effect meta-analysis will be conducted to estimate the pooled effect of sustainment strategies separately by setting (i.e. clinical and community). Sub-group analyses will be undertaken to explore possible causes of statistical heterogeneity and may include the following: time period, single or multi-strategy, type of setting, and type of intervention. Differences between sub-groups will be statistically compared.

**Discussion/conclusion:**

This will be the first systematic review to determine the effect of strategies designed to support sustainment on sustaining the implementation of EBIs in clinical and community settings. The findings of this review will directly inform the design of future sustainability-focused implementation trials. Further, these findings will inform the development of a sustainability practice guide for public health practitioners.

**Systematic review registration:**

PROSPERO CRD42022352333.

## Background

### Global burden of chronic disease

Preventable chronic diseases such as heart disease, diabetes, and respiratory disease account for a significant proportion of morbidity and mortality, attributing to 70% of all deaths internationally [1, 2]. There are several key behavioural risk factors associated with the development of chronic diseases across the life course including the following: physical inactivity, poor diet, harmful alcohol use, and tobacco smoking [3, 4]. Each of these behavioural risk factors is responsible for a considerable proportion (2.78–9.24%) of the total disease burden globally [5].

The World Health Organization (WHO) recommends the implementation of evidence-based interventions (EBIs) in clinical (e.g. hospitals, general practitioner (GP) surgeries, dental practices, community health centres, and charity-based health programmes or initiatives) and community settings (e.g. schools, early childcare services, sporting clubs/organisations, and community centres) to target and reduce the prevalence and severity of these behavioural risk factors [4]. The routine and widespread implementation of EBIs (e.g. targeting physical activity [6] and alcohol reduction [7]) to address the prevention of chronic disease in these settings is important, as they provide centralised points of access to reach a large proportion of the population, and they have existing infrastructure to support intervention delivery [8]. Consequently, there have been substantial investments made by governments internationally in the development and implementation of EBIs to address behavioural risk factors for chronic disease in these settings [9–11].

There are two distinct outcomes within the field of sustainability: ‘sustainability’ and ‘sustainment’. There are several definitions in the literature for both sustainment and sustainability [12–14]. For this review, we make a clear distinction between sustainment and sustainability. We view sustainment as an outcome, defined by Damschroder et al. (2022) as ‘the extent the innovation is in place or being delivered long-term’ [15]. Sustainability is defined by Moore et al. (2017) as ‘after a defined period of time, the program, clinical intervention, and/or implementation strategies continue to be delivered and/or individual behaviour change (i.e., clinician, patient) is maintained; the programme and individual behaviour change may evolve or adapt while continuing to produce benefits for individuals/systems’ [13]. Recent research argues that sustainability should be viewed as a dynamic process with interventions updated according to new evidence and adapted to meet the changing needs of the context and population in which it is being delivered [12, 16].

Although many EBIs provide significant benefits when initially implemented, the effects of these interventions often diminish once initial implementation support or resources are withdrawn, and consequently, the quality of intervention delivery decreases or is discontinued entirely [17]. Therefore, long-term positive health impacts are often not realised [17–20] or are not achieved equitably across a range of settings and populations [21]. Further, discontinuation of programmes may also have important implications for wasted investments in time and resources, as well as community member and practitioner mistrust and wariness to engage in future implementation efforts [21]. Even with multi-level implementation support and significant financial investment, ‘initiative decay’ is common [10]. For example, a systematic review by Wiltsey Stirman and Kimberly [17], focusing on the sustainment of public health and clinical interventions, found that out of 125 studies included in the review, the majority of interventions were only partially sustained (i.e. continuation of some, but not all elements of the intervention), following full initial implementation. Overall, less than half of the interventions included in this review were sustained to high levels of fidelity. Another recent systematic review by Herlitz and MacIntyre [20], which aimed to determine the sustainment of school-based public health interventions, found that of the 18 included interventions, none continued to be delivered in their entirety (i.e. all components) once initial implementation support (start-up funding and/or other resources) had been withdrawn.

Accordingly, policymakers are increasingly concerned with the sustainability of EBIs and highlight the importance of ensuring the sustained delivery of such interventions long term. To ensure that the positive effects of EBIs continue and health impact is realised, the public health investment in initial implementation is not wasted, and that community support, trust, and engagement with such interventions are not lost; it is vital that the implementation of these EBIs be sustained [21].

### What impacts on sustaining EBIs

Understanding the determinants of sustainment is essential to successfully design effective sustainment support strategies and reduce implementation decline [22]. Theoretical frameworks, such as the Dynamic Sustainability Framework [12], the Program Sustainability Assessment Tool [23], and the Integrated Sustainability Framework [16], identify and categorise a range of factors that may impact the sustainment of EBIs. In general, most frameworks identify sustainability determinants at multiple levels, that is, the salient outer contextual factors (e.g. external funding environment), inner contextual factors (e.g. programme champions in the organisation), processes (e.g. strategic planning), intervention characteristics (e.g. fit with context and population), and implementer characteristics (e.g. staff attitude, motivation, and skills). Further, systematic reviews of determinants to sustaining EBIs in specific clinical and community settings have identified a number of factors perceived by stakeholders. The most frequently identified being as follows: the availability of equipment, resources and facilities, continued executive or leadership support, and staff turnover [17, 19, 20, 22]. Moreover, there are commonalities in factors that commonly impact sustainability across both clinical and community settings such as funding and external partnerships, organisational factors (e.g. alignment with values, needs, resources, and priorities of the organisation) and support (e.g. the presence of programme champions, leadership support), and practitioner/workforce characteristics (e.g. staff motivation and attitudes [16]. The information gathered from these reviews can be utilised to determine which factors to prioritise when developing strategies to sustain EBI delivery.

### The need for effective strategies to support sustainment

If policymakers and practitioners are to address determinants of sustaining EBIs, it is important to determine which strategies are most effective in supporting sustainment. It is also important to note that strategies designed to support sustainment may overlap with strategies designed to support initial implementation. While there is a growing body of evidence regarding the effectiveness of strategies to support the initial implementation of EBIs [24–26], to our knowledge, only one review has aimed to collate strategies designed specifically to support sustainment [27].This review of strategies used within community-based settings found only six studies that reported the use of nine types of strategies designed to support sustainment. The most commonly reported strategies were funding and/or contracting for EBIs, continued use, and maintenance of workforce skills through continued training, booster training sessions, supervision, and feedback. However, the review was descriptive and, given the low number of studies conducted to date, did not synthesise any data relating to the effectiveness or impact of the strategies designed to support sustainment. Additionally, as this review only focused on community settings, there is a current gap which presents a need to synthesise strategies designed to support sustainment in a broader range of settings. Consequently, the field is bereft of an evidence base of effective strategies for sustainment. Research within sustainability science is rapidly increasing. Consequently, there are likely to be numerous new studies that may provide evidence of effective strategies designed to support sustainment. Therefore, the primary aim of this review is to determine the effect of strategies aiming to sustain the chronic disease prevention initiatives targeting key health behaviours (i.e. physical inactivity, poor diet, harmful alcohol use, and tobacco smoking) in clinical and community settings.

The secondary aims of this review are as follows:

1. Examine the effectiveness of strategies designed to support sustainment on relevant health outcomes (including physical activity, healthy eating, obesity prevention, smoking cessation, or harmful alcohol use).
2. Describe the cost implications of strategies designed to support sustainment.
3. Identify if there are any unintended/adverse effects of strategies designed to support sustainment on end users.

## Methods

This systematic review protocol was registered with PROSPERO on 20 August 2022 (Registration ID: CRD42022352333) and is reported in accordance with the Preferred Reporting Items for Systematic review and Meta-Analysis Protocols checklist (PRISMA-P) [28].

### Eligibility criteria

#### Types of studies

We will include any randomised study with a control group that aims to assess the effect of a strategy or group of strategies to sustain the implementation of a chronic disease prevention EBI in a clinical or community setting. We will include the following types of studies:

- Randomised controlled trial (RCT) (with a parallel control group)
- Cluster randomised controlled trial (C-RCT) (with a parallel control group and at least two clusters randomised to each group)
- Stepped-wedge trial
- Cross over (only data prior to crossover will be used in the analysis)

We will restrict the review to this set of designs for pragmatic reasons due to the size of this review. Further, these designs are considered as gold standard for assessing casual effects, so are most appropriate for addressing the research questions. We will only include studies that compare a strategy or group of strategies to improve sustainment of a physical activity, healthy eating, obesity prevention, smoking cessation, or harmful alcohol use EBI (also termed as policy, practice, or program) with no sustainment intervention or ‘usual practice’. There will be no restriction on the length of the study follow-up period due to the varied definitions of sustainment within the literature. There will also be no restriction on country of origin or language. However, we will exclude studies that are not focused on assessing the effect of a sustainment strategy on the sustained implementation of a policy, practice, or programme as a specific aim.

#### Types of participants

We will include managers, policy makers, staff, clinicians, or volunteers delivering, or supporting the delivery of, EBIs to patients in clinical settings including hospitals, GP surgeries, community health centres, and charity-based health programmes or initiatives (e.g. charity-run smoking cessation and healthy eating interventions in low socioeconomic countries/areas).

We will also include managers, policy makers, staff, or volunteers delivering, or supporting the delivery of, EBIs to end users in community settings including educational settings (i.e. primary and secondary schools, colleges, and universities), childcare services (long day care, family day care, preschools, and nurseries), elite or nonelite sports organisations and clubs (professional and amateur sports clubs, sporting governing bodies), and community centres (youth centres, community outreach centres).

#### Types of interventions

We will include any study that employs a strategy or group of strategies with the explicit aim of sustaining the implementation of a smoking cessation, healthy eating, physical activity, alcohol or obesity prevention policy, practice, or programme by usual staff, clinicians, or volunteers within the setting, for example managers, policy makers, nurses, doctors, teachers, and carers. Studies embedding principles of sustainability into strategies that have a primary aim of increasing adoption or implementation of EBIs will be excluded. Strategies designed to support sustainment will be classified based on the sustainability-explicit expert recommendations for implementing change (ERIC) glossary [29]. To be eligible, strategies designed to support sustainment must be distinct from continuous quality improvement (CQI). Distinctions will be made between sustainment and CQI by recognising CQI as studies focused on making immediate improvements to an individual organisation [30]. This is compared to sustainability trials which are typically designed based on theoretical frameworks or models and focused on making generalisable improvements, rather than being restricted on one individual organisation.

### Types of outcome measures

#### Primary outcome measures

Studies that report on any objective or subjective measure of the sustainment of a health prevention policy, practice, or programme within any of the eligible settings will be included. This may include the ongoing delivery of physical activity, dietary, alcohol, or smoking cessation interventions in line with public health or clinical guidelines.

Sustained implementation must be a measure of usual staff or volunteer delivery of the policy, practice, or programme and not be externally supported by research personnel, except for the purposes of data collection. Individual outcomes such as sustained effects of patient’s participation in a programme (e.g. their participation in a healthy eating programme) are not considered sustainment outcomes.

#### Secondary outcome measures

Data on secondary outcomes will only be extracted for those studies that first meet the eligibility criteria for the primary review outcomes. For example, if a study aims to sustain the implementation of a physical activity policy practice, but reports on dietary outcomes and physical activity practices, only data regarding physical activity practices will the extracted.

Secondary outcomes include the following:

1. Health outcomes where an EBI or initiative is used to target modifiable health behaviour risks related to chronic disease. I.e. any objective or subjective measure of diet (e.g. fruit/vegetable intake), physical activity (e.g. minutes of physical activity during the school day), sedentary behaviour (e.g. daily minutes of sedentary time), weight status (e.g. BMI (body mass index)), alcohol consumption (e.g. number of standard drinks consumed on a typical drinking day), and smoking cessation (e.g. weekly number of cigarettes smoked). A hierarchy will be used to prioritise multiple measures of the same health outcome.
2. Cost outcomes relating to estimates of absolute costs, the assessment of the cost-effectiveness, or budget impact of strategies designed to support sustainment.
3. Any reported adverse effects of strategies designed to support sustainment. This may include negative impact on health outcomes (e.g. an increase in injury rates following physical activity initiatives), disruption to service operation or staff attitudes (e.g. negative impact on staff motivation or cohesion), or negative consequences to other key programmes or practices (e.g. lack of funding for other vital programmes due to reallocation of funding).

### Search methods for identification of studies

We will conduct searches for peer-reviewed articles in relevant electronic databases. 

### Electronic searches

We will conduct searches in the following electronic databases: the Cochrane Central Register of Controlled trials (CENTRAL) (2022) via Cochrane Library; MEDLINE (1946 to November, 2022), PsycINFO (1950 to November, 2022), and Embase (1947 to November, 2022) via OVID; CINAHL (November, 2022) via EBSCO; and SCOPUS (November, 2022) and Education Research Complete (November, 2022) via EBSCO.

#### Search strategy/search terms

Search terms will be developed based on reviews conducted by Shelton et al. [16] (maintenance/sustainability) and Wolfenden et al. [9–11] (physical activity, nutrition, and obesity, implementation, and setting) and will cover the following four concepts:

1. Sustainability (other terms include maintenance, durability, continuation, institutionalisation, routinization, normalisation, integration, adherence)
2. Heath behaviours (e.g. physical activity, healthy eating, smoking cessation)
3. Clinical settings (e.g. hospitals, general practice)
4. Community settings (e.g. schools, workplaces, community centres)

### Data collection and analysis

#### Selection criteria

The search results from the electronic databases will be managed and duplicates identified using EndNote. The de-duplicated library will be imported into Covidence software, where article screening will occur. Both title and abstract and full-text screening will be conducted independently by two members of the research team, who will assess study eligibility according to the inclusion criteria. Any conflicts will be resolved by consensus. In instances where the study eligibility cannot be resolved via consensus, a third review author will make the final decision.

#### Data extraction and management

Two review authors unblinded to author and journal information will independently extract information from the included studies. We will record the information extracted from the included studies in a data extraction form, developed based on the recommendations of the Cochrane Public Health Group Guide for Developing a Cochrane Protocol [31]. The data extraction form will be piloted before the initiation of the review. Data extraction discrepancies between review authors will be resolved by consensus or by a third review author if required.

We will extract the following information:

1. Study eligibility as well as the study design, date of publication, EBI, country, the demographic/socioeconomic characteristics of the programme and participants, the number of experimental conditions, setting, overall study duration, and time points measured.
2. Characteristics of the strategy designed to support sustainment, including strategy description and duration of initial implementation support and length of time since withdrawn (if noted), duration of strategies (i.e. duration for which the sustainment strategy was in place), description of strategies, the theoretical underpinning of the strategy (if noted in the study), process evaluation measures (e.g. acceptability and appropriateness), and information to allow classification against the sustainability-explicit ERIC glossary [29]. Strategies will be described in line with the sustainability-explicit ERIC glossary [29].
3. Primary and secondary outcomes within each study, including the data collection method, validity of measures used, effect size, and measures of outcome variability
4. Source(s) of research funding and potential conflicts of interest

### Assessment of risk of bias in included studies

#### Overall risk of bias

Two review authors will assess risk of bias independently for each review outcome using Version 2 of the Cochrane risk-of-bias tool for randomised trials (RoB 2) described by Sterne et al. [32]. Signalling questions will be used for the following domains: Bias arising from the randomisation process, bias due to deviations from intended interventions, bias due to missing outcome data, bias in measurement of the outcome, bias in the selection of the reported result, and overall bias. The response options to the signalling questions will be as follows: ‘yes’, ‘probably yes’, ‘probably no’, ‘no’, and ‘no information’. Once the signalling questions are answered, a risk-of-bias judgement and one of three levels (low risk of bias, some concerns, or high risk of bias) will be assigned to each domain. Stepped wedge trials will be assessed for risk of bias using RoB 2, with consideration given to time confounding. Crossover trials will be assessed using the RoB 2 extension for crossover designs, and only the initial segment prior to crossover will be used in the analysis. We will use the ROB2 extension for cluster trials for the assessment of the risk of bias for cluster RCTs, which includes consideration of the following additional domains: recruitment bias, baseline imbalances, loss of clusters, incorrect analysis, contamination, and compatibility with individually randomised trials. An overall risk of bias will be assigned to each study outcome giving consideration to all of the above domains. Overall risk of bias for study outcomes will be assessed against set criteria and judged as follows: ‘low risk of bias’ (‘the trial is judged to be at low risk of bias for all domains’), ‘some concerns’ (the trial is judged to raise some concerns in at least one domain, but not be at high risk of bias for any domain), and high risk of bias (the trial is judged to be at high risk of bias in at least on domain OR the trial is judged to have some concerns for multiple domains in a way that substantially lowers confidence in the result) (Higgins et al., 2022). The risk of bias of the included studies will be documented in a ‘risk-of-bias’ table.

### Synthesis methods

Study characteristics will be grouped as types of studies, participants (i.e. clinical or community), and strategies designed to support sustainment. Strategies designed to support sustainment will be classified using the sustainability-explicit ERIC glossary [29]. The sustainability-explicit ERIC glossary is a taxonomy which categorises and defines strategies designed to support sustainment. It is an adapted version of the original ERIC [33], which has been extended with a specific focus on sustainability. The sustainability-explicit ERIC glossary will allow us to code the strategies in this review based on the standardised definitions included in the glossary. Deductive and inductive coding approaches will be used, and any strategies that do not fit within the sustainability-explicit ERIC glossary will be added. The effect of interest will be intention to treat, and we will prioritise differences between groups at follow-up, rather than differences between groups in the change from baseline. Primary outcomes will be reported using odds ratios, and any primary outcomes measured as means and standard deviations will be transformed into odds ratios (Higgins, et al., 2022). For secondary outcomes, the most appropriate effect type will be used, which will include odds ratios for dichotomous outcomes and means for continuous outcomes. Random-effects meta-analyses will be undertaken to estimate a pooled treatment effect overall for the primary outcome and by health behaviour for secondary outcomes (i.e. physical activity, alcohol consumption, dietary outcomes, and tobacco use). If we are unable to conduct a meta-analysis due to insufficient or incomplete data (e.g. missing standard deviations) that cannot be estimated from the data reported by authors, we will synthesise results using vote counting based on the direction of effect [31], with such methods reporting in compliance with the synthesis without meta-analysis (SWIM) guidelines [34]. For trials with multiple follow-up periods, we will use data from the final follow-up period reported. For studies that report multiple results for primary and secondary outcomes, we will prioritise the most objectively measured. Results from cluster- and individual-level RCTs will be combined. The standard error from cluster trials that do not adjust for clustering will be adjusted for unit of analysis errors following recommended procedures outlined by the Cochrane Handbook [31]. Trials reporting multiple, relevant intervention arms will be combined into a single group following methods outlined in the Cochrane Handbook [31].

### Sensitivity analyses

Where there are sufficient studies, a sensitivity analyses removing studies with high risk of bias will be undertaken. If imputation of intra-class correlation coefficient (ICC) values to adjust for clustered trials is required, a sensitivity analysis assessing different ICC values will also be conducted.

### Assessing heterogeneity and subgroup analyses

Statistical heterogeneity will be assessed by reviewing the distribution of studies on the forest plots and assessing the *I*^2^ statistic. Pre-specified sub-group analyses will be undertaken to explore possible causes of statistical heterogeneity and will include time period classified as sustainability and type of setting (i.e. clinical or community). Differences between sub-groups will be statistically compared following procedures recommended by the Cochrane Handbook; within subgroup differences will not be interpreted.

## Discussion

This systematic review will synthesise current evidence on the effect of strategies designed to support sustainment of chronic disease prevention policies, practices, and programmes. This will be the first systematic review to determine the effect of strategies designed to support the sustainment of EBIs in both clinical and community settings. The findings of this review will directly inform the design of future sustainability and implementation trials. Further, these findings will help inform the development of a sustainability practice guide for public health practitioners. The main limitation of this review protocol is our restriction to only RCTs. In focusing exclusively on RCTs, we may overlook valuable insights from alternative study designs, such as quasi-experimental and qualitative methods, which offer a more nuanced understanding of real-world constraints and pressures. Future reviews may wish to broaden the included study types which could capture important information on the effect of sustainment strategies. Further, while our review will use data from included studies final follow-up period, the inclusion of longitudinal data could offer valuable insights into the temporal dynamics of sustainment strategy effectiveness and provide a more nuanced understanding of how interventions unfold over time. Therefore, we recommend that future reviews consider incorporating multiple follow-up times.

## Data Availability

Data and materials relating to this review are available from the corresponding author on reasonable request.

## Abbreviations

EBIs: Evidenced-based interventions
PRISMA: Preferred Reporting Items for Systematic review and Meta-Analysis
CQI: Continuous quality improvement
BMI: Body mass index
RCT: Randomised controlled trial
C-RCT: Cluster randomised controlled trial
ICC: Intra-class correlation coefficient
